# A prediction model‐based algorithm for computer‐assisted database screening of adverse drug reactions in the Netherlands

**DOI:** 10.1002/pds.4364

**Published:** 2017-12-21

**Authors:** Joep H.G. Scholl, Florence P.A.M. van Hunsel, Eelko Hak, Eugène P. van Puijenbroek

**Affiliations:** ^1^ Netherlands Pharmacovigilance Centre Lareb 's‐Hertogenbosch The Netherlands; ^2^ PharmacoTherapy, ‐Epidemiology and ‐Economics, Groningen Research Institute of Pharmacy University of Groningen The Netherlands

**Keywords:** adverse drug reaction, pharmacoepidemiology, pharmacovigilance, prediction model, signal detection

## Abstract

**Purpose:**

The statistical screening of pharmacovigilance databases containing spontaneously reported adverse drug reactions (ADRs) is mainly based on disproportionality analysis. The aim of this study was to improve the efficiency of full database screening using a prediction model‐based approach.

**Methods:**

A logistic regression‐based prediction model containing 5 candidate predictors was developed and internally validated using the Summary of Product Characteristics as the gold standard for the outcome. All drug‐ADR associations, with the exception of those related to vaccines, with a minimum of 3 reports formed the training data for the model. Performance was based on the area under the receiver operating characteristic curve (AUC). Results were compared with the current method of database screening based on the number of previously analyzed associations.

**Results:**

A total of 25 026 unique drug‐ADR associations formed the training data for the model. The final model contained all 5 candidate predictors (number of reports, disproportionality, reports from healthcare professionals, reports from marketing authorization holders, Naranjo score). The AUC for the full model was 0.740 (95% CI; 0.734–0.747). The internal validity was good based on the calibration curve and bootstrapping analysis (AUC after bootstrapping = 0.739). Compared with the old method, the AUC increased from 0.649 to 0.740, and the proportion of potential signals increased by approximately 50% (from 12.3% to 19.4%).

**Conclusions:**

A prediction model‐based approach can be a useful tool to create priority‐based listings for signal detection in databases consisting of spontaneous ADRs.

## 1 INTRODUCTION

Spontaneous reporting systems have been the cornerstone of pharmacovigilance since their introduction in the 1960s. The main aim of spontaneous reporting is the early detection of previously unrecognized adverse drug reactions (ADRs). In addition, spontaneous reporting can also be useful for obtaining information on new aspects of known associations between drugs and ADRs.^1^ Although spontaneous reporting has its methodological shortcomings from an epidemiological perspective, it is a valuable method for the early detection of ADRs.^2^, ^3^ An effective signal detection process is a key element of a spontaneous reporting system in pharmacovigilance. For the detection of signals, the Netherlands Pharmacovigilance Centre Lareb has historically relied on a case‐by‐case clinical review of incoming reports, directly submitted by health care professionals (HCP) and consumers. This review is performed by trained pharmacovigilance assessors, the majority of them being medical doctors and pharmacists. Reports that may represent a potential signal in the view of the assessor are discussed in a weekly scientific meeting. Potential signals undergo a more detailed analysis.^4^

Lareb has criteria in place for assessors to determine which reports should be discussed at the weekly scientific meeting. However, because multiple assessors are involved in this process and the selection of reports for the weekly scientific is prone to some level of subjectivity, a computer‐assisted database screening tool is in place as an additional approach to reduce the risk for missing potential signals.^5^ The screening tool is even more important for ADRs reported by marketing authorization holders (MAHs) that may be indicative of potential signals, as these are not assessed on a case‐by‐case basis at Lareb.

The computer‐assisted database screening tool used in the Netherlands relies on the number of reports of drug‐ADR associations and disproportionality based on the reporting odds ratio (ROR). With the disproportionality analyses, the observed rate of a drug and ADR together is compared with an expected value based on their relative frequencies reported individually in the spontaneous reporting database.^5^, ^6^ In the approach applied at our centre, the lower limit of the 2‐sided 95% confidence interval (CI) is used combined with a number of at least 3 reports per association. Associations can be automatically selected by the screening tool based on 1 or more of the following pre‐defined criteria; Anatomical Therapeutic Chemical code (allowing the assessor to screen more efficiently), ADR being unlabeled in the Summary of Product Characteristics (SPC), number of reports (≥3), threshold of the lower limit of the 2‐sided 95% CI of the ROR (ROR025) (>1), pre‐specified calendar date, set during previous analysis. Associations highlighted by the screening tool undergo a short analysis by trained pharmacovigilance assessors. Based on the decision of the assessor, subsequent new thresholds can be specified (ADR, unlabeled, number of reports or lower limit 95% CI, new date) or the association can undergo further detailed analyses. The association will be highlighted again as soon as one of the aforementioned criteria is met.^7^ Although the current approach facilitates the selection of potential signals, the downside of this approach is that it yields a high number of associations that need an initial, short analysis, which is a time‐consuming process. With the current methods, associations can be ranked on the basis of number of reports or the level of disproportionality. However, it is not possible to prioritize based on other, possibly relevant features of the reported association. Prioritization based on associations that would theoretically yield the number of highest potential signals is seen by Lareb as a way to improve timelines of the signal detection process.

The Uppsala Monitoring Centre, WHO Collaborating Centre for International Drug Monitoring, has developed a data‐driven screening algorithm for emerging drug safety signals that accounts for report quality and content, called vigiRank.^8^ VigiRank is a model which uses several predictive values as determined in the WHO Global ICSR database; VigiBase®. Some of the predictors that were found for this model are not applicable for a national database, such as geographical spread. The Lareb database contains a high number of reports with free text and have a relatively high documentation grade, as represented by the vigiGrade® completeness score of the Lareb reports in VigiBase®.^9^ Because Lareb does a case‐by‐case analyses of all reports, except those received through the MAH, it is known for each association whether the ADR is labeled in the Dutch SPC. Also, for each report (except those received through the MAH), a causality score (Naranjo) is calculated.^10^ Based on this, and other, additional information that is available for ICSRs, a more elaborate set of predictors would probably be suited for a screening tool on the Dutch national spontaneous database.

The primary aim of this study was to develop a new prediction model‐based screening tool in order to improve statistical signal detection. Secondary aim was to compare this new model to the old screening tool, which is based on the number of reports and the ROR025.

**KEY POINTS**

- Current methods for full database screening of ADRs are mainly based on disproportionality, which has its limits due to its sensitivity for several types of selection bias.
- We developed a prediction model‐based approach to generate a priority list of drug‐ADR associations to be analyzed.
- The performance of the model and the comparison with the current method showed that the prediction model‐based approach is to be preferred over the current method.

## 2 METHODS

### 2.1 Setting

In this study, we developed a logistic regression‐based prediction model for drug‐ADR associations present in the Lareb spontaneous reporting database. Using the linear predictor of this model, a prioritized list of associations not present in the SPC was made for comparison with the current method. The data for this study were derived from the database of the Netherlands Pharmacovigilance Centre Lareb. This database consists of spontaneous reports of suspected ADRs reported to Lareb directly by both HCP and consumers. Additionally, reports from MAHs regarding events that occurred in The Netherlands are imported into our database from the European Medicines Agency database Eudravigilance. Each report contains 1 or more drug‐ADR associations. For the development of the prediction model, all drug‐ADR associations were extracted from each report. ADRs were coded using the preferred terms from the Medical Dictionary for Regulatory Activities.^11^ Drugs were classified according to the WHO Anatomical Therapeutic Chemical classification system.^12^

### 2.2 Outcome

The outcome of the model was defined as the presence in the SPC of each unique drug‐ADR association at the time of the analysis. Although the use of the SPC to determine if an association is actually an ADR (implying causality) has its limitations, it has been used in several studies aimed at statistical signal detection.^13^, ^14^ At Lareb, for each association present in an ICSR received directly from a HCP or consumer, a causality assessment using the Naranjo score is performed.^10^ For Naranjo question 1: “Are there previous conclusive reports on this reaction?”, 3 options are available at our centre: 1) “Yes, listed in SPC”, 2) “Yes, described in other literature”, 3) “No/unknown”. In order to be in line with the original Naranjo scale, a score of +1 is assigned if option 1) or 2) is chosen and a score of 0 if option 3) is chosen in routine practice at Lareb. For the purpose of this study, associations with the outcome were defined as option 1), and associations without the outcome as option 2) or 3). Associations consisting of MAH reports only were manually assessed for the presence in the SPC.

### 2.3 Inclusion / exclusion criteria

All reports received until 12‐May‐2016 were considered eligible for inclusion with the exception of reports related to vaccines. For these reports, a method other than Naranjo is used to determine causality. Because that particular method lacks information about the presence in the SPC, reports related to vaccines were excluded. For statistical considerations, only associations with a minimum of 3 reports were selected, because this was deemed to be the minimum number of reports needed for a reliable ROR estimation.

### 2.4 Selection of candidate predictors

For each association, the following variables were selected as candidate predictors in the model:

1. The number of ICSRs.
2. The lower limit of the 2‐sided 95%CI of the ROR (ROR025).
3. The percentage of ICSRs derived from health care professionals (HCP). This variable was selected because HCP reports differ from consumer reports.^15^
4. The percentage of ICSRs derived from MAHs. This variable was selected because it is our experience that MAH reports differ from reports received directly from HPCs / consumers, most likely due to regulatory obligations for MAHs. This is confirmed by the fact that only 0.2% of the reports from MAHs are included in signals published by Lareb (for HCPs and consumers this is 3.9% and 2.0%, respectively^16^).
5. The mean Naranjo score across all reports containing the association. The answer to question 1 was excluded in the calculation of the Naranjo score because this question is the basis for the outcome variable of the prediction model. Additionally, because no Naranjo scores are present for MAH reports, scores for those reports were set at +2 because we considered it valid to assume that the ADR occurred after the suspect drug was given (Naranjo question 2).

### 2.5 Development of the model

A multivariable logistic regression model was developed using backward step‐wise selection. The training data consisted of all drug‐ADR associations with a minimum of 3 reports. The candidate predictors with *P* < 0.05 (Wald test) were fitted into the model. Due to non‐linearity of the predictors, they were converted to categorical variables with equally sized categories. For all candidate predictors, the generalized variance inflation factor, a measure of variance for categorical variables, was calculated to investigate collinearity, using 4 as a conservative cut‐off value.^17^, ^18^

### 2.6 Evaluation of the model

Internal validation of the model was performed by means of a calibration curve of the observed versus predicted probabilities, Hosmer‐Lemeshow goodness of fit testing, and bootstrap resampling. For the latter, the validate function from the R package “rms” was used (number of bootstrap samples = 1000, no backward step‐down variable deletion). The performance of the model was based on the area under the receiver operating characteristic curve (AUC).

Because our prediction model was developed to be used specifically for the Lareb database, external validation was not considered to be relevant. Additionally, previous signal detection research showed that there are substantial differences among different pharmacovigilance databases,^19^ making validation using an external data source (eg, a database from another country) of limited value.

### 2.7 Comparison with current method of signal detection

Although the current method of screening at Lareb is not based on a prediction model, we considered it of interest to compare the current model with a model containing only the number of reports and the ROR025, which are the basis for the current screening method. The comparison consisted of 2 elements: First, the performance in terms of the AUC was compared between both methods. Second, we constructed priority lists of associations not present in the SPC, containing the first 10% and 20% of the associations, respectively. The priority list was based on the value of the linear predictor from the old and new model, respectively. With the list, we determined the (relative) number of associations that had been previously analyzed in depth (mainly triggered by case‐by‐case assessment), as a proxy for a possible signal, for both methods. This second method was performed to investigate whether a theoretical difference in performance, based on the AUC, also resulted in a different amount of potentially interesting signals in practice.

## 3 RESULTS

### 3.1 Descriptive statistics and model development

A total of 151 033 ICSRs, containing 120 171 unique drug‐ADR associations were extracted from the database. After the selection of associations with a minimum of 3 ICSRs, 25 026 associations remained as the training data for fitting of the model. Of these, 17 071 associations (68.2%) were present in the SPC. Additional information is presented in Table 1. Median values with interquartile ranges (IQR) and proportions of the candidate predictors are shown in Table 2.

**Table 1:** Descriptive statistics of the ICSRs used for analysis

	Number (*n*)
Number of ICSRs	151 033
Number of associations	341 478
Number of unique associations (total)	120 171
Number of unique associations (*n* ≥ 3)	25 026
Present in SPC	17 071 (68.2%)
Number of unique drugs^a^	1745
Number of unique suspected ADRs^b^	5726

a Classified according to the Anatomical Therapeutic Chemical (ATC) classification system.

b Coded as Medical Dictionary for Regulatory Activities preferred terms.

**Table 2:** Descriptive statistics of the candidate predictors used in the analysis

Candidate Predictor	Associations Listed in SPC (*n* = 17 071)	Associations not Listed in SPC (*n* = 7955)
Number of ICSRs per association (median / IQR)	5 (7)	4 (3)
ROR025 per association (median / IQR)	1.3 (3.1)	1.3 (5.3)
Naranjo score per association (median / IQR)	1.8 (0.7)	2.0 (0.4)
Associations from MAH reports (%)	57.4	42.6
Associations from HCP reports (%)	86.8	13.2

Abbreviations: IQR, interquartile range; ROR025, lower limit of the 2‐sided 95%CI of the ROR.

Prior to the regression analysis, all candidate predictors were divided into 4 categories of equal sizes due to non‐linearity. After the backward step‐wise selection procedure, all candidate predictors were included into the final model. VIF values for the assessment of multicollinearity were below the pre‐defined threshold (4) for all predictors in the model. The proportion of associations present in the SPC increased with increasing numbers of reports and an increasing proportion of HCP reports. For ROR025, Naranjo score and proportion of MAH reports the direction of the effect was less consistent. For ROR025, an increase in category was associated with increasing coefficients except for the highest category. For both Naranjo and MAH reports, the lower categories were associated with a higher coefficient (see also Table 3).

**Table 3:** Full multivariable model with model parameters and measure for multicollinearity (VIF)

Predictor	Number of Observations	Regression Coefficient	Standard Error	*P*‐value	VIF
Intercept		−0.09	0.07	0.23	
Number of ICSRs per association (*n*)					1.50
3	7859	Reference category			
4–5	7008	0.34	0.04	<0.0001	
6–8	4197	0.72	0.05	<0.0001	
>8	5962	1.22	0.05	<0.0001	
ROR025 per association					1.63
≤0.54	6365	Reference category			
0.55–1.29	6181	0.33	0.04	<0.0001	
1.30–4.18	6224	0.52	0.04	<0.0001	
>4.18	6256	0.39	0.04	<0.0001	
Naranjo score per association					2.12
0–1.33	6709	Reference category			
1.34–1.94	5805	0.07	0.05	0.16	
1.95–2.00	6784	−0.39	0.05	<0.0001	
>2.00	5728	−0.11	0.04	0.01	
Percentage of MAH reports					2.36
0%	10 071	Reference category			
0.1–20.0%	2711	0.64	0.07	<0.0001	
20.1–75.0%	6323	0.07	0.04	0.14	
>75.0%	5921	−0.79	0.07	<0.0001	
Percentage of HCP reports					2.47
0–12.5%	6305	Reference category			
12.6–50.0%	7130	0.41	0.06	<0.0001	
50.1–75.0%	5654	0.48	0.06	<0.0001	
>75.0%	5937	0.70	0.07	<0.0001	

Abbreviations: ROR025, lower limit of the 2‐sided 95%CI of the ROR; VIF, variance inflation factor.

### 3.2 Performance and validation

**Figure 1:**
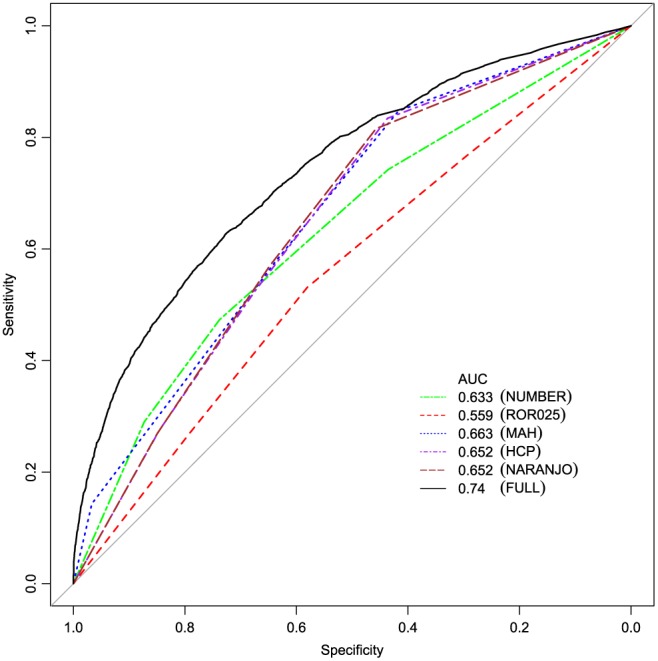
Receiver operating characteristics (ROC) curves for the various models. AUC, area under the curve; NUMBER, number of reports; ROR025, lower limit of the 2‐sided 95% CI of the ROR; MAH, percentage of marketing authorization holder reports; HCP, percentage of reports by health care professionals; Naranjo, Naranjo score; FULL, full model [Colour figure can be viewed at wileyonlinelibrary.com]

The performance of the model was satisfactory, based on the area under the receiver operating characteristic curve (AUC = 0.740; 95%CI 0.734–0.747; see Figure 1). The 3 strongest predictors in the model were more than 8 reports per association, followed by a percentage of HCP reports of 75% or higher, and a percentage of MAH reports between 0% and 20% (see Table 3).

**Figure 2:**
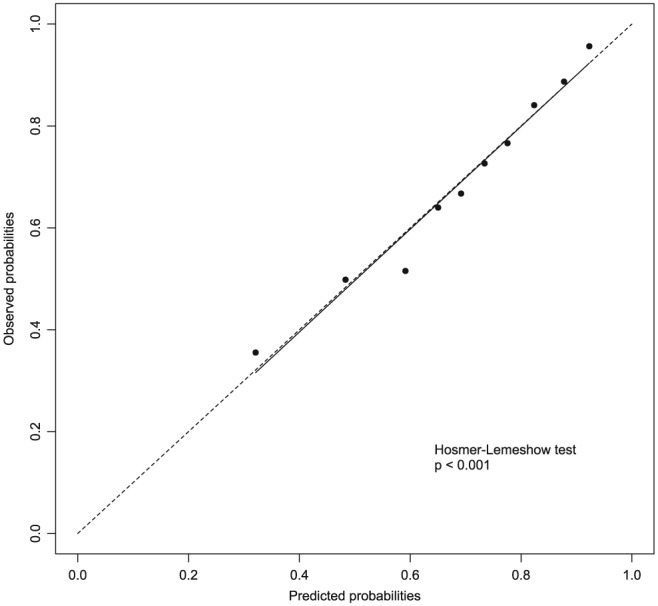
Calibration curve of the final model. The dotted line represents a perfect calibration

The calibration curve of the model shows good calibration based on the observed versus predicted probabilities (see Figure 2). Additionally, bootstrap resampling showed only a marginal difference in the AUC of the model (AUC after bootstrapping = 0.739), indicating no overfitting of the model.

### 3.3 Comparison with current method

**Figure 3:**
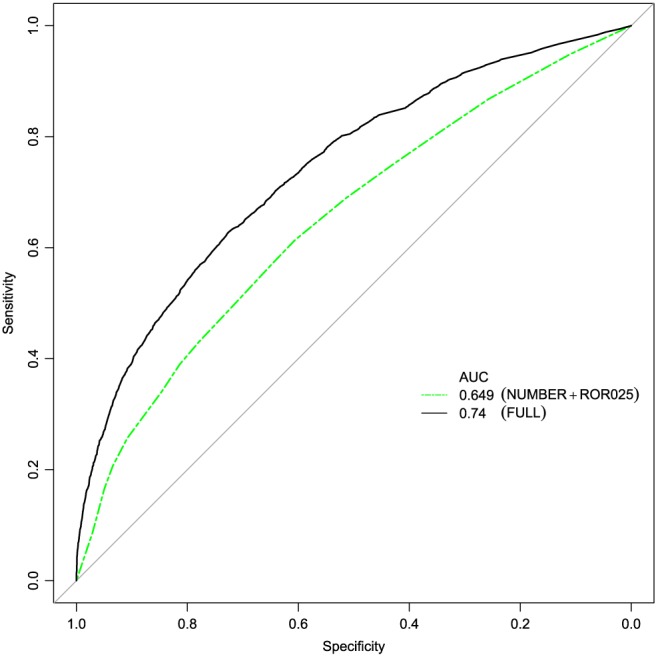
Receiver operating characteristics (ROC) curves for the new model (FULL) and the old model (NUMBER+ ROR025). AUC, area under the curve; NUMBER, number of reports; ROR025, lower limit of the 2‐sided 95% CI of the ROR [Colour figure can be viewed at wileyonlinelibrary.com]

A comparison of the new model with the current method based on a model with only the number of reports and ROR025 as predictors showed an increased performance (AUC_new_ = 0.740; AUC_old_ = 0.649; see Figure 3).

As mentioned previously, this is a theoretical comparison, because the current method used at Lareb is not based on a prediction model. Therefore, the models were not compared in terms of their AUCs, but priority lists of associations were made for both models, and these were compared in terms of possible signals. The results of these analyses show that the proportion of possible signals increased by 58.2% (from 12.3% to 19.4%) and 44.2% (from 9.6% to 13.9%) depending on the number of associations used (800 vs 1600, respectively). Additional information is present in Table 4.

**Table 4:** Performance of the new prediction model in terms of possible signals

	Number of Possible Signals (*n*/%)		Relative Increase (%)
	Old Model	New Model	
Top‐800^a^	98 (12.3)	155 (19.4)	58.2
Top‐1600^a^	154 (9.6)	222 (13.9)	44.2

a *Refers to the first 800 (10%) and 1600 (20%) associations not listed in the SPC of the linear predictor‐based priority lists for each model, respectively*.

## 4 DISCUSSION

In this study, we developed a prediction model‐based screening tool aimed at improving statistical signal detection of our spontaneous ADR reports. Five relevant characteristics (number of reports, disproportionality, Naranjo score, proportion MAH reports, proportion HCP reports) were chosen as potential predictors in the model. For Naranjo, we considered to use the scoring (doubtful, possible, probable, definite) for categorization. However, this would result in a skewed distribution of observations due to the (very) low numbers of associations with outcome “definite”. Our choice to set the Naranjo score for all MAH reports to +2 was based on the assumption that the ADR occurred after the suspect drug was given, which gives a score of +2. Because no case‐by‐case assessment is performed for MAH reports at our centre, other items from the Naranjo algorithm could not be answered. This approach could lead to less diversity in the Naranjo scores, and as a result impact the performance of the model. However, a recent pilot study performed at our centre investigating case‐by‐case analysis of MAH reports showed that the documentation level of the reports was often poor.^20^ Therefore, availability of information on additional Naranjo items would most likely be limited. Although important in statistical signal detection,^13^, ^21^ the time to onset was not included as a predictor in our model. In a previous study, we investigated the values of the time to onset in statistical signal detection and found that it was of limited value in the investigated setting.^22^ Overall, the model performed well (AUC = 0.740) and showed good calibration. The highly significant result of the Hosmer‐Lemeshow test (*P* < 0.001), indicating a poor goodness of fit, is most likely explained by the large sample size and should be interpreted with caution.^23^ Based on the calibration curve and the bootstrap resampling, the internal validation was considered satisfactory. The latter showed a negligible difference in AUC (0.740 versus 0.739).

We found little differences in AUC values among individual predictors, although disproportionality (ROR025) seems to have the lowest predictive value. This may be explained by the fact that disproportionality is sensitive to selective reporting and other types of bias.^24^, ^25^, ^26^

Within subgroups of predictors, we found some noteworthy results regarding the regression coefficients. The predictors “number of reports” and “percentage of HCP reports” showed a consistent increase in coefficients with increasing categories, which was as expected. For ROR025 and Naranjo, we anticipated similar results, which was not the case though (see Table 3). This may be explained by the fact that the outcome used in our model (presence of the association in the SPC) does not by definition imply causality because true ADRs are not necessarily present in this document, and on the other hand, events that are not true ADRs may be present. Additionally, the categories used for Naranjo in the model limit the conclusions that can be drawn from the regression coefficients. For fitting of the model, 4 more or less equally sized categories were made due to non‐linearity of the parameter. As a result, the first 3 categories range from 0 to 2, and the fourth category contains all values above 2, whereas the actual Naranjo score has a range from −4 till 13. For the percentage of MAH reports, there seems to be an inverse relationship with presence in the SPC. One striking difference between MAH reports and reports received directly from HCPs or consumers that we found in our previously described pilot study^20^ was the presence of ADRs in MAH reports that are not truly ADRs but, for example, outcomes (eg, death, hospitalization, lack of efficacy) or events related to the indication of the drug (eg, terminal state in cancer patients with metastases). The fact that these types of events are not likely to be present in the SPC and have a low percentage of reports directly reported to Lareb may be an explanation for the inverse relationship.

Although one can debate the validity of the use of the SPC as the gold standard for causality, we considered it to be the most comprehensive and up‐to‐date data source with information regarding ADRs that is publicly available. A different approach could have been the method used for vigiRank, where a set of historical safety signals was used as a reference set of positive controls.^8^ However, the issue regarding causality and presence in the SPC as mentioned earlier remains, because they used the SPC to define the negative controls. We considered it more appropriate to use the same gold standard for both the positive and negative controls and therefore decided to use the SPC for both. Additionally, the use of a reference set would most likely result in a less heterogeneous set of ADRs because it would probably not contain more common ADRs (eg, headache, dizziness, nausea, etc.), although these types of ADRs are among the most frequently present in spontaneous reports.

The linear predictor‐based priority lists comparing the old and new model showed a substantial increase in potential signals among the most highly ranked drug‐ADR combinations not present in the SPC. In this context, the increase in potential signals should be seen in terms or earlier detection due to prioritization and not in terms of signals that would, or would not be picked up by either method.

Previous research suggests that results obtained from signal detection algorithms depend on the database the algorithm is applied to.^8^, ^19^ The same will hold for our algorithm. For example, in the Netherlands, we receive a substantial amount of ICSRs reported by patients, but this is not necessarily the case in other countries. Therefore, the use of the amount of HCP reports as a candidate predictor may not be a logical choice for other databases. Consequently, the development of such a model should be based on the reporting and database characteristics of the country or region it is applied to. Nevertheless, the method of generating a prediction model‐based priority list of signals could be useful in other (spontaneous reporting) databases.

One of the limitations of our study is the risk of bias due to selective reporting. Because the database contains well‐established associations, it is reasonable to assume that these associations are reported more frequently than unknown associations, therewith influencing the predictors in the model. In an alternative approach, the values of the predictors immediately prior to the recognition of the association could be used in the model. However, recovering the date of recognition for several thousand associations may prove to be infeasible.

In conclusion, this study shows that a prediction model‐based screening tool can be used to generate priority‐based listings of drug‐ADR associations for signal detection. Additionally, as seen in other studies,^8^, ^27^ the introduction of variables other than the number of reports and disproportionality can increase screening efficiency due to priority‐based assessment of drug‐ADR associations.

## ETHICS STATEMENT

The authors state that no ethical approval was needed.

## CONFLICT OF INTEREST

The authors declare no conflict of interest related to this work

## References

[1] Practical Aspects of Signal Detection in Pharmacovigilance: Report of CIOMS Working Group VIII. Geneva: 2010.

[2] Raine JM . Risk Management: A European Regulatory View. New York: Wiley; 2007.

[3] Lester J , Neyarapally GA , Lipowski E , Graham CF , Hall M , Dal PG . Evaluation of FDA safety‐related drug label changes in 2010. Pharmacoepidemiol Drug Saf. 2013;22(3):302‐305. 23280652 10.1002/pds.3395

[4] van Hunsel F , Talsma A , van Puijenbroek E , de Jong‐van den Berg L , van Grootheest K . The proportion of patient reports of suspected ADRs to signal detection in the Netherlands: case‐control study. Pharmacoepidemiol Drug Saf. 2011;20(3):286‐291. 21351310 10.1002/pds.2092

[5] Wisniewski AF , Bate A , Bousquet C , et al. Good signal detection practices: evidence from IMI PROTECT. Drug Saf. 2016;39(6):469‐490. 26951233 10.1007/s40264-016-0405-1 PMC4871909

[6] Stricker BH , Tijssen JG . Serum sickness‐like reactions to cefaclor. J Clin Epidemiol. 1992;45(10):1177‐1184. 1474414 10.1016/0895-4356(92)90158-j

[7] van Hunsel F , Ekhart C . Experiences with a computer‐assisted database screening tool at The Netherlands Pharmacovigilance Centre Lareb. Pharmacoepidemiol Drug Saf. 2015;24(S1):442

[8] Caster O , Juhlin K , Watson S , Noren GN . Improved statistical signal detection in pharmacovigilance by combining multiple strength‐of‐evidence aspects in vigiRank. Drug Saf. 2014;37(8):617‐628. 25052742 10.1007/s40264-014-0204-5 PMC4134478

[9] Bergvall T , Noren GN , Lindquist M . vigiGrade: a tool to identify well‐documented individual case reports and highlight systematic data quality issues. Drug Saf. 2014;37(1):65‐77. 24343765 10.1007/s40264-013-0131-x PMC6447519

[10] Naranjo CA , Busto U , Sellers EM , et al. A method for estimating the probability of adverse drug reactions. Clin Pharmacol Ther. 1981;30(2):239‐245. 7249508 10.1038/clpt.1981.154

[11] Brown EG , Wood L , Wood S . The medical dictionary for regulatory activities (MedDRA). Drug Saf. 1999;20(2):109‐117. 10082069 10.2165/00002018-199920020-00002

[12] Structure and Principles of the ATC classification system. http://www whocc no/atc/structure_and_principles/, 2011 (Accessed April 3, 2013).

[13] van Holle L , Zeinoun Z , Bauchau V , Verstraeten T . Using time‐to‐onset for detecting safety signals in spontaneous reports of adverse events following immunization: a proof of concept study. Pharmacoepidemiol Drug Saf. 2012;21(6):603‐610. 22383219 10.1002/pds.3226

[14] Szarfman A , Machado SG , O'Neill RT . Use of screening algorithms and computer systems to efficiently signal higher‐than‐expected combinations of drugs and events in the US FDA's spontaneous reports database. Drug Saf. 2002;25(6):381‐392. 12071774 10.2165/00002018-200225060-00001

[15] Rolfes L , van Hunsel F , Wilkes S , van Grootheest K , Van Puijenbroek EP . Adverse drug reaction reports of patients and healthcare professionals‐differences in reported information. Pharmacoepidemiol Drug Saf. 2015;24(2):152‐158. 25079444 10.1002/pds.3687

[16] van Hunsel F , de Waal S , Harmark L . The contribution of direct patient reported ADRs to drug safety signals in the Netherlands from 2010 to 2015. Pharmacoepidemiol Drug Saf. 2017;26(8):977‐983. 28524293 10.1002/pds.4236

[17] Fox J , Monette G . Generalized collinearity diagnostics. J Am Stat Assoc. 1992;87(417):178‐183.

[18] Neter J , Kutner MH , Nachtsheim CJ , Wasserman W . Applied linear statistical models. Irwin; 1996.

[19] Candore G , Juhlin K , Manlik K , et al. Comparison of statistical signal detection methods within and across spontaneous reporting databases. Drug Saf. 2015;38(6):577‐587. 25899605 10.1007/s40264-015-0289-5

[20] Scholl JH , van Hunsel F , Harmark L . Case‐by‐case assessment of ICSRs from marketing authorization holders: a pilot study. Drug Saf. 2015;38(10):986 Ref Type: Abstract

[21] Norén GN , Hopstadius J , Bate A , Star K , Edwards IR . Temporal pattern discovery in longitudinal electronic patient records. Data Min Knowl Discov. 2010;20(3):361‐387.

[22] Scholl JH , Van Puijenbroek EP . The value of time‐to‐onset in statistical signal detection of adverse drug reactions: a comparison with disproportionality analysis in spontaneous reports from the Netherlands. Pharmacoepidemiol Drug Saf. 2016;25(12):1361‐1367. 27686554 10.1002/pds.4115

[23] Hosmer DW , Hosmer T , Le CS , Lemeshow S . A comparison of goodness‐of‐fit tests for the logistic regression model. Stat Med. 1997;16(9):965‐980. 9160492 10.1002/(sici)1097-0258(19970515)16:9<965::aid-sim509>3.0.co;2-o

[24] Bate A , Evans SJ . Quantitative signal detection using spontaneous ADR reporting. Pharmacoepidemiol Drug Saf. 2009;18(6):427‐436. 19358225 10.1002/pds.1742

[25] Maignen F , Hauben M , Hung E , van Holle L , Dogne JM . Assessing the extent and impact of the masking effect of disproportionality analyses on two spontaneous reporting systems databases. Pharmacoepidemiol Drug Saf. 2014;23(2):195‐207. 24243665 10.1002/pds.3529

[26] Hartnell NR , Wilson JP . Replication of the Weber effect using postmarketing adverse event reports voluntarily submitted to the United States Food and Drug Administration. Pharmacotherapy. 2004;24(6):743‐749. 15222664 10.1592/phco.24.8.743.36068

[27] van Holle L , Bauchau V . Use of logistic regression to combine two causality criteria for signal detection in vaccine spontaneous report data. Drug Saf. 2014;37(12):1047‐1057. 25395263 10.1007/s40264-014-0237-9 PMC4243000

